# Evaluating the impact of cannabis use on thalamic connectivity in youth at clinical high risk of psychosis

**DOI:** 10.1186/s12888-015-0656-x

**Published:** 2015-11-09

**Authors:** Lisa Buchy, Tyrone D. Cannon, Alan Anticevic, Kristina Lyngberg, Kristin S. Cadenhead, Barbara A. Cornblatt, Thomas H. McGlashan, Diana O. Perkins, Larry J. Seidman, Ming T. Tsuang, Elaine F. Walker, Scott W. Woods, Carrie E. Bearden, Daniel H. Mathalon, Jean Addington

**Affiliations:** Department of Psychiatry, University of Calgary, Calgary, Alberta Canada; Department of Psychology, Yale University, New Haven, CT USA; Department of Psychiatry, Yale University, New Haven, CT USA; Department of Neuroscience, Faculty of Science, University of Calgary, Alberta, Canada; Department of Psychiatry, UCSD, La Jolla, CA USA; Department of Psychiatry, Zucker Hillside Hospital, Long Island, NY USA; Department of Psychiatry, University of North Carolina, Chapel Hill, NC USA; Department of Psychiatry, Harvard Medical School at Beth Israel Deaconess Medical Center and Massachusetts General Hospital, Boston, MA USA; Departments of Psychology and Psychiatry, Emory University, Atlanta, GA USA; Departments of Psychiatry and Biobehavioral Sciences and Psychology, UCLA, Los Angeles, CA USA; University of California, San Francisco, San Francisco, CA USA; Mathison Centre for Mental Health Research and Education, University of Calgary, 3280 Hospital Drive NW, Calgary, Alberta Canada T2N 4Z6

**Keywords:** Cannabis, Clinical high risk, Functional connectivity, Resting state functional magnetic resonance imaging, Psychosis, Schizophrenia, Thalamus

## Abstract

**Background:**

Disruptions in thalamic functional connectivity have been observed in people with schizophrenia and in youth at clinical high risk (CHR) of psychosis. However, the impact of environmental risk factors for psychosis on thalamic dysconnectivity is poorly understood. We tested whether thalamic dysconnectivity is related to patterns of cannabis use in a CHR sample.

**Methods:**

162 CHR and 105 control participants were assessed on cannabis use severity, frequency, and age at onset of first use as part of the North American Prodrome Longitudinal Study and completed resting-state fMRI scans. Whole-brain thalamic functional connectivity maps were generated using individual subjects’ anatomically defined thalamic seeds.

**Results:**

Thalamic connectivity did not significantly correlate with current cannabis use severity or frequency in either CHR or controls. In CHR cannabis users, a significant correlation emerged between attenuated thalamic connectivity with left sensory/motor cortex and a younger age at onset of cannabis use. CHR who used cannabis before age 15 did not differ on thalamic connectivity as compared to CHR who used after age 15 or CHR who were cannabis naïve. No group differences in thalamic connectivity emerged when comparing CHR separated by moderate/high use frequency, low-frequency or cannabis naïve.

**Conclusions:**

Although a younger age at onset of cannabis use may be associated with disrupted thalamo-cortical coupling, cannabis use does not appear to be an identifying characteristic for thalamic connectivity in CHR with moderate/high use frequency compared to low-frequency users or CHR who are cannabis naïve.

**Electronic supplementary material:**

The online version of this article (doi:10.1186/s12888-015-0656-x) contains supplementary material, which is available to authorized users.

## Background

Schizophrenia is a neurodevelopmental disorder characterized by disturbed connectivity between components of large-scale brain networks [1]. One of the most profoundly altered neural systems involves thalamo-cortical networks, which have been characterized by hypo-connectivity between the thalamus and prefrontal cortex (PFC) [2] and cerebellum [3, 4], and thalamic hyper-connectivity with somatosensory and motor areas [2, 3, 5, 6]. A very recent study [7] in CHR youth observed a pattern of thalamic dysconnectivity similar to that seen in schizophrenia, that was more severe in those who later converted to schizophrenia. In addition, both patterns of thalamic dysconnectivity significantly correlated with attenuated positive symptom severity. This series of reports suggests that aberrant thalamic modulation of information to and from cortical foci occurs prior to the onset of psychosis and may in part underlie the pathophysiology of psychotic illnesses. However, the degree to which these functional brain alterations are impacted by well-known risk factors for psychosis is poorly understood.

Cannabis use is one environmental factor that is known to modify the risk for psychosis. It is the most widely used illicit substance in schizophrenia and its risk states [8], and epidemiological data suggests a role for cannabis in the onset of psychosis [9–12]. Recent prospective data in youth at CHR for psychosis has indicated that, among lifetime cannabis users, higher baseline use severity [13] and frequency [14], and first use before the age of 15 [14, 15] are all associated with an increase in conversion to psychosis. Although no studies have evaluated the relation between cannabis use patterns and functional connectivity in CHR, two studies have reported thalamic volume loss in association with cannabis use in youth at genetic high risk of schizophrenia. One study reported that frequent cannabis use was associated with third ventricle enlargement, likely reflecting gray matter loss in the adjacent anterior medial region of the thalamus [16]. This research group later published 2-year longitudinal data showing that cannabis exposed individuals at genetic high risk of schizophrenia showed bilateral thalamic volume loss compared to a non-exposed group [17]. Given that cannabis use is known to impact longitudinal thalamic structure in people with a genetic high risk for schizophrenia, this raises the question about the impact of cannabis use patterns on thalamic functional connectivity in high risk samples. The relationship between thalamic neural circuitry with prefrontal and cerebellar regions and cannabis use may be particularly relevant, as post-mortem studies have identified these regions as being rich in cannabinoid (CB1) receptors [18].

In summary, the research to date suggests that thalamic functional abnormalities are present in schizophrenia and its risk states, that thalamic structure is impacted by lifetime cannabis use exposure and frequency in high risk samples, and that early age of first use and greater use severity and frequency heighten the risk for psychosis. In a prior study in the current sample [7], resting state functional magnetic resonance imaging (rs-fMRI) thalamic connectivity analyses revealed 5 regional clusters that showed abnormal connectivity in CHR individuals relative to healthy controls: lateral PFC, anterior cingulate gyrus, cerebellum, and left and right sensory/motor cortices. These regions share topographically organized reciprocal connections with the thalamus, are rich in CB1 receptors (see above), and have been linked to structural alterations in cannabis users (see above). Therefore, in the current study we tested the hypotheses that in individuals at CHR for psychosis, thalamic dysconnectivity in these previously identified [7] regional clusters are associated with 1) severity and frequency of cannabis use, 2) age at onset of cannabis use, 3) current cannabis use vs. no use, 4) early-onset cannabis use (i.e. first use before age 15) compared to late-onset use (first use at or after age 15), cannabis naïve CHR and controls, and 5) cannabis use in CHR high-frequency users compared to moderate- or low-frequency users, CHR who are abstinent, and controls. If such links were established, it could provide evidence that CHR youth are indeed particularly vulnerable to the brain structural and functional consequences of cannabis use.

## Methods

### Participants

All participants were recruited as part of the multi-site North American Prodrome Longitudinal Study (NAPLS 2) [20], which was established to investigate predictors and mechanisms of conversion to psychosis. The final NAPLS sample consists of 764 CHR participants and 280 healthy controls. This paper reports on the 162 CHR and 105 healthy controls who provided baseline resting-state fMRI scans and also completed a detailed baseline assessment on cannabis use. All CHR participants were required to meet the Criteria of Prodromal Syndromes (COPS) using the Structured Interview for Prodromal-Risk Syndromes (SIPS) [21]. Participants were excluded if they met criteria for any current or lifetime axis I psychotic disorder, IQ < 70, past or current history of central nervous system disorder or DSM-IV criteria for current substance dependence disorder. Control participants were also excluded if they had a first degree relative with a current or past psychotic disorder. We made every attempt to match groups on age, sex and parental socioeconomic status. A more detailed description of ascertainment, inclusion and exclusion criteria, and participant details is provided elsewhere [20].

### Measures

The SIPS and the Scale of Prodromal Symptoms (SOPS) [21] were used to assess criteria for a prodromal syndrome and severity of attenuated positive symptoms. Post-training agreement on determining the prodromal diagnoses was excellent (kappa = 0.90) [20].

Cannabis and other drug use was rated with the Alcohol and Drug Use Scale [22] which records severity (1 = abstinent, 2 = use without impairment, 3 = abuse, 4 = dependence) and frequency of use (0 = no use, 1 = once or twice per month, 2 = 3-4 times per month, 3 = 1–2 times per week, 4 = 3–4 times per week, 5 = almost daily) in the last month. Based on commonly used measures and interview questions in the literature [12, 15, 23] we also enquired the age at which cannabis was first used.

### Cannabis groups

Given evidence that first cannabis use before the age of 15 confers greater risk for conversion to psychosis [14, 15], we separated CHR participants into three groups of cannabis users: early-onset (cannabis use before age 15), late-onset (cannabis use after age 15), and cannabis naïve (had never used cannabis).

Secondly, given evidence that higher cannabis use frequency confers greater risk for psychosis [14], participants were separated into four groups according to their frequency of cannabis use: high-frequency (i.e. daily users), moderate-frequency (1–4 times per week), low-frequency (1–4 times per month) and abstinent. However, only one CHR subject was using daily (see Table 1); therefore this individual was combined with the moderate-frequency group to form a moderate/high-frequency group.

**Table 1:** Demographic and clinical characteristics of the CHR and healthy control groups

	CHR *n* = 162	HC *n* = 105		
	n (%)	n (%)	χ ^2^	*p*-value
Sex				
Male	96 (59)	57 (54)	0.62	0.43
Female	66 (41)	48 (46)		
Race				
First Nations	2 (1)	2 (2)	10.1	0.26
Asian	9 (6)	10 (10)		
Black	35 (22)	28 (27)		
Latin America/Middle East/White	93 (57)	59 (55)		
Inter-racial	23 (14)	6 (6)		
AUS/DUS cannabis use frequency				
Abstinent	112 (69)	92 (88)	17.1	0.004
1–4 times per month	26 (16)	11 (10)		
1–4 times per week	21 (13)	2 (2)		
Almost daily	3 (2)	0 (0)		
AUS/DUS cannabis use severity				
Abstinent	116 (72)	92 (88)	11.7	0.003
Use without impairment	37 (23)	13 (12)		
Abuse	9 (6)	0 (0)		
	Mean (SD)	Mean (SD)	*t*	*p*-value
Age (years)	19.4 (4.21)	19.5 (4.62)	0.25	0.80
Education (years)	11.8 (2.50)	12.7 (3.46)	2.32	0.02
Age at onset of cannabis use (years)^a^	16.1 (3.20)	16.0 (2.30)	0.25	0.81

Note. *CHR* Clinical High Risk, *HC* Healthy Controls, *SD* Standard Deviation

^a^Information on age of onset of cannabis use was available for 99 CHR and 38 control participants

### Neuroimaging data acquisition

Scanning was performed at eight sites. Five sites (UCLA, Emory, Harvard, UNC, and Yale) used Siemens-Trio 3 T scanners, two sites (Zucker-Hillside Hospital and UCSD) used GE HDx scanners, and one site (Calgary) used a GE Discovery scanner. Previously, we reported on results in which 8 subjects each travelled to all 8 NAPLS-2 sites and were scanned twice on successive days [24]. Results indicated that site-related variations were trivial across scanners, suggesting that neuroimaging can be performed across multiple sites at the same level of reliability as at a single site. All neuroimaging and functional connectivity analyses followed prior work and best practices in the clinical connectivity literature with details presented in Additional file 1.

### Seed-based functional connectivity analysis (rs-fcMRI) based on thalamic anatomy

Our seed-based fcMRI approach followed prior studies using anatomically-defined thalamic seeds [25]. In-house Matlab tools [26, 27] were used to examine thalamus coupling with all gray matter voxels. We computed a seed-based thalamus correlation map by extracting average time-series across all voxels in each subject’s bilateral thalamus anatomically defined through Freesurfer-based segmentation [28, 29]. This entire thalamic signal was then correlated with each gray matter voxel, and the computed Pearson correlation values were transformed to z-scores using a Fisher r-to-Z transform, providing a map for each subject that were entered into 2^nd^-level analyses where each voxel’s value represented its connectivity with the whole thalamus. We restricted analyses to five regions that revealed robust thalamic dysconnectivity in a sample of chronic SCZ patients [30] and that were similarly abnormal when identified using a priori thalamic masks in the NAPLS-2 sample of CHR youth [7] whose data are further analyzed in the current study: right lateral PFC, anterior cingulate gyrus, cerebellum, and left and right sensory/motor cortices, as shown in Figure 1a and 1b of reference [7]. Regional coordinates are shown in Table 2. The r-to-z transformed values were averaged across all voxels within each of these regions.

**Table 2:** Partial correlations between resting state thalamic networks and AUS/DUS rated cannabis use severity, frequency, and age at onset of cannabis use in the CHR and healthy control groups

X	Y	Z	Hemisphere	Anatomical landmark	Severity		Frequency		Age at onset of cannabis use	
					CHR	HC	CHR	HC	CHR	HC
					*n* = 162	*n* = 105	*n* = 162	*n* = 105	*n* = 99	*n* = 38
−31	−67	−43	Left	Cerebellum	0.01 (0.96)	0.01 (0.99)	−0.01 (0.89)	0.04 (0.83)	0.21 (0.04)	0.13 (0.47)
31	42	31	Right	Lateral prefrontal cortex	0.09 (0.28)	−0.16 (0.36)	0.14 (0.09)	−0.23 (0.19)	0.18 (0.08)	0.11 (0.55)
2	21	36	Midline	Anterior cingulate gyrus	−0.03 (0.68)	−0.08 (0.65)	−0.04 (0.63)	−0.08 (0.65)	0.16 (0.16)	0.20 (0.25)
−35	−23	58	Left	Sensory/ motor cortex	0.03 (0.73)	−0.06 (0.74)	−0.02 (0.79)	−0.07 (0.69)	**0.26 (0.01)**	0.10 (0.58)
19	−27	70	Right	Sensory/ motor cortex	0.03 (0.75)	0.12 (0.50)	−0.01 (0.87)	0.02 (0.91)	0.23 (0.03)	0.10 (0.59)

Note. *AUS/DUS* Alcohol and drug use scale. *CHR* Clinical high risk, *HC* healthy control. Age, alcohol use and tobacco use have been entered as covariates. Results expressed as Pearson’s correlations with corresponding p-value in brackets. Significant results are bolded

### Statistical analysis

Chi-square analyses for categorical variables and t-tests for continuous variables were used to compare CHR and control groups on demographics. To evaluate the impact of confounding variables, Spearman correlations were used to evaluate the relationship between thalamic volumes and functional connectivity and age, alcohol use and tobacco use. Variables that significantly correlated with thalamic connectivity were entered as covariates in subsequent analyses. Specifically, partial correlations were used to measure associations of cannabis use severity, frequency and age at onset of use with thalamic functional connectivity. CHR early-onset, late-onset and cannabis naïve groups were compared on thalamic connectivity across all 5 regions using a two-way repeated measures MANCOVA with regions as the dependent measure and demographic and clinical variables as covariates. To follow-up on significant Group X Region interaction effects, separate one-way ANOVAs were performed with Tukey’s post-hoc tests to compare groups where appropriate. All F-tests involving repeated measure factors were based on Wilks’ Lambda. Similarly, two-way repeated measures MANCOVA was used to compare CHR use frequency groups (moderate/high-frequency, low-frequency, abstinent) on thalamic connectivity with regions as the dependent variable and demographic and clinical variables as covariates. To follow-up on significant Group X Region interaction effects, separate one-way ANOVAs were performed with Tukey’s post-hoc test applied where appropriate. The critical p-value was set to 0.01 following Bonferroni correction for multiple comparisons for the five regions of interest when conducting follow up tests on individual regions. NAPLS participants who did not provide cannabis ratings were not considered in any analyses. Statistical analyses were conducted using SPSS 21.0.

### Procedures

All eight sites involved in this longitudinal study of predictors of conversion to psychosis recruited CHR individuals. Raters were experienced research clinicians who demonstrated adequate reliability at routine reliability checks. Gold standard post-training agreement on the critical threshold for determining initial eligibility based on the SIPS was excellent (kappa = 0.90). The principal investigator or clinical psychiatrist or psychologist at each site conducted a comprehensive clinical assessment to determine if entry criteria were met. JA chaired weekly conference calls to review criteria for all individuals admitted to the study. Clinical assessments that included the Alcohol and Drug Use Scale (AUS/DUS) and the SOPS were conducted at baseline.

Informed written consent was obtained from those who met criteria and were judged fully competent to give consent. Parental consent was obtained from parents/guardians of participants who were under age 16. Consent was a written consent signed by the participant, study coordinator and witness. Participants receive a copy of the consent. The participants in this study were not patients, and they do not have a diagnosis. They are young people who are presenting with signs that suggest they may be at risk for developing psychosis. None of the participants have symptoms that would lead to any diagnosis of a DSM-IV psychotic disorder. All of the participants have IQ > than 70. All participants were reviewed with either a licenced psychiatrist or licensed clinical psychologist at all of the sites. The study was carried out in accordance with The Code of Ethics of the World Medical Association (Declaration of Helsinki) for experiments involving humans and approved by the Institutional Review Boards of all eight NAPLS sites: Emory: Emory Institutional review Board; Zucker Hillside: North-Shore LIJ Health System Office of the Human Research Protection Program; Yale: Yale University Human Investigations Committee; UCLA: UCLA Institutional review Board; UCSD: UCSD Human Research Protections Program; Calgary: University of Calgary Conjoint Health Research Ethics Board; UNC: University of North Carolina Biomedical IRB; Harvard: Committee on Clinical Investigation.

## Results

### Demographics

Demographic, clinical and behavioral characteristics of CHR and control subjects are summarized in Table 1. Age significantly and positively correlated with thalamic connectivity with the anterior cingulate cortex (*p* = 0.01) but no other region of interest (all ps > 0.06). Males and females did not differ on thalamic connectivity with any region of interest (all ps > 0.47). Alcohol use severity significantly and inversely correlated with thalamic connectivity with anterior cingulate cortex (p = 0.04) but no other region (all ps > 0.22). Tobacco use severity significantly and positively correlated with thalmo-cerebellar connectivity (*p* = 0.03) but no other region of interest (all ps > 0.33). Therefore, age, alcohol use and tobacco use were entered as covariates in all statistical analyses.

The CHR and control participants did not differ on age, sex or ethnicity, however, controls had significantly higher education. Groups did not differ on age at onset of cannabis use, though CHR participants had significantly higher severity and frequency of cannabis use.

Thalamic volumes did not significantly correlate with thalamic connectivity with any region (all r’s < 0.05, all p’s > 0.20).

### Thalamic functional connectivity in relation to cannabis use severity and frequency

To test hypothesis 2, we evaluated correlations between thalamic connectivity and cannabis use severity and frequency, controlling for age, tobacco and alcohol use. These results are shown in Table 2. In CHR, no significant correlations emerged between cannabis use severity or frequency and thalamic connectivity in any region. Similarly, in controls, no significant correlations were observed between cannabis use severity or frequency and thalamic functional connectivity.

### Thalamic functional connectivity in relation to age at onset of cannabis use

To test hypothesis 3, we examined correlations between thalamic connectivity with age at onset of cannabis use in CHR and controls, entering age, tobacco and alcohol as covariates.

As shown in Table 2, in CHR a significant correlation was seen between thalamic hyper-connectivity with left sensory/motor cortex with a younger age at onset of cannabis use. Trends toward hyper-connectivity between the thalamus and right sensory motor cortex, and thalamo-cerebellar hypo-connectivity, in association with a younger age at onset of cannabis use were also seen. In the control group, no significant correlations were observed between age at onset of cannabis use and thalamic connectivity.

### Thalamic functional connectivity in current cannabis users compared to non-users

To test hypothesis 1, we evaluated the thalamic functional connectivity in current cannabis users compared to non-users using a two-way repeated measures MANCOVA with age, alcohol and tobacco as covariates. In CHR, 46 were current users and 116 were not currently using cannabis. The Group effect was non-significant, F(1, 157) = 0.34, *p* = 0.56. The Group X Region interaction was also non-significant, F(4, 154) = 1.11, *p* = 0.35, Wilkes Lambda = 0.97, indicating that CHR currently using cannabis did not differ from those currently abstinent from cannabis on thalamic connectivity in any region of interest.

In controls, 13 were current users and 92 were not currently using cannabis. The two-way repeated measures MANCOVA indicated a non-significant Group effect, F(1, 100) = 0.14, *p* = 0.91. The Group X Region interaction was also non-significant, F(4, 97) = 0.84, *p* = 0. 15, Wilkes Lambda = 0.97, indicating that controls currently using cannabis did not differ from those currently abstinent from cannabis on thalamic connectivity in any region of interest.

### Early age at onset of cannabis use and thalamic connectivity in CHR and controls

To test hypothesis 4, we evaluated the association between age at onset of cannabis use and thalamic connectivity in CHR and controls. Within the CHR group, 32 people had an early-onset use of cannabis, 67 had a late-onset, and 61 were cannabis naïve. Demographic and clinical characteristics of these groups are summarized in Additional file 2. These groups significantly differed in age, education, SOPS total positive symptoms, as well as current alcohol, tobacco and cannabis use severity. Therefore, these variables were included as covariates in the MANOVA.

When comparing these three CHR groups and controls on thalamic network dysconnectivity, the two-way repeated measures MANOVA indicated a non-significant Group effect, F(3, 254) = 0.62, *p* = 0.60. The Group X Region interaction was also non-significant, F(12, 664.4) = 0.69, *p* = 0.76, Wilkes Lambda = 0.97.

### Comparison of cannabis use frequency groups on thalamic functional connectivity in CHR and controls

To test hypothesis 5, we evaluated thalamic connectivity as a function of cannabis use frequency groups in CHR, and controls. Demographic and clinical characteristics of these groups are summarized in Additional file 3. Within the CHR group, 24 had moderate/high-frequency cannabis use, 26 people had a low-frequency use, and 112 were abstinent. These groups significantly differed on sex, SOPS total positive symptoms, as well as current alcohol, tobacco and cannabis use severity. Therefore, these variables were included as covariates in the MANOVA.

The two-way repeated measures MANOVA indicated a non-significant Group effect, F(3, 258) = 0.80, *p* = 0.50. The Group X Region interaction effect was also non-significant, F(12, 675.0) = 1.48, *p* = 0.13, Wilkes Lambda = 0.93.

## Discussion

Schizophrenia and its risk states have been characterized by dysconnectivity in thalamic neural circuitry [2–7]. However, the impact of cannabis use—one environmental factor thought to increase the risk for psychosis—on thalamic connectivity remains unknown. In the current study we used resting state fMRI to characterize thalamic dysconnectivity in relation to patterns of cannabis use in a CHR sample and relative to a healthy control group. Results indicated that thalamic connectivity did not significantly correlate with current cannabis use severity or frequency in either group. A younger age at onset of cannabis use was significantly correlated with thalamic hyper-connectivity with left sensory/motor cortex in CHR participants, but not in controls. CHR early-onset users, late-onset users and cannabis naïve participants were indistinguishable on thalamic neural circuitry. No group differences in thalamic connectivity were seen between CHR separated by moderate/high-frequency, low-frequency or abstinent. These results indicate that although a younger age at onset of cannabis use correlated with disruptions in thalamo-cortical connectivity in CHR, in the group analyses, CHR early-onset cannabis users showed similar patterns of connectivity to both late-onset and cannabis naïve CHR. Moderate/high-frequency of cannabis use does not appear to be an identifying characteristic for thalamic connectivity in CHR.

The severity or frequency of current cannabis use did not correlate with thalamic connectivity in our CHR or control participants. Although no published studies have reported on the neural correlates of current cannabis use severity in a CHR sample, higher frequency of cannabis use over 1 year has been associated with decreased PFC volumes in subjects with an at-risk mental state [31]. Findings from a cross-sectional study in people with a genetic risk of schizophrenia suggests that higher cannabis use frequency correlated with greater 3^rd^ ventricular volumes [16]. Individuals with adolescent onset schizophrenia who used cannabis three times a week for at least 6 months have a documented widespread gray matter density loss and decreased white matter structural connectivity as measured with diffusion tensor imaging [32]. Although the existing literature suggests that people at genetic risk for schizophrenia and people with schizophrenia are particularly vulnerable to brain volume loss with greater exposure to cannabis, the current results suggest that in people at CHR of psychosis thalamic neural circuitry is not affected by higher severity or usage rates of cannabis.

Perhaps the most novel finding is the significant correlation that emerged between increased thalamic connectivity with left sensory/motor cortex and a younger age at onset of cannabis use in CHR participants. The sensory/motor cortex is rich in CB1 receptors [33, 34] and cannabis intake has been associated with volumetric reductions in frontal or superior parietal cortices in a CHR sample [31], and early-onset schizophrenia cohort [35] and in first-episode schizophrenia [36]. Longitudinal data suggests that adolescent cannabis users with schizophrenia and a comorbid cannabis use disorder show an atypical pattern of cortical development over an 18-month period compared to schizophrenia patients without a cannabis disorder [37]. Interestingly, separating CHR participants into early-onset, late-onset and naïve cohorts did not reveal an age-specific effect of onset of cannabis use on thalamic connectivity. This set of results may suggest that thalamic connectivity is impacted by the age of exposure of cannabis in a linear fashion rather than by exposure during a sensitive period in development in people at CHR of psychosis. These results also suggest that cannabis use before age 15 is not an identifying characteristic for thalamic functional dysconnectivity in CHR youth [7].

Current cannabis use did not appear to reflect on thalamic connectivity in our CHR or control samples. The only other study to conduct a cross-sectional evaluation of the neural correlates of lifetime cannabis use in a high risk sample reported that genetic high risk individuals with heavy lifetime cannabis use (i.e., ≥40 times across the lifetime) showed decreased total cortical thickness compared to moderate users and those who were cannabis naïve [38]. In first-episode schizophrenia, cannabis use has been linked to decreased gray matter density in posterior cingulate cortex, an area high in CB1 receptor expression [36], but not to cerebellar volumes [39], an area also dense in CB1 receptors. Current cannabis use in people with an at-risk mental state has also been linked to decreased volumes of posterior as well as anterior cingulate cortices [40]. Interestingly, two longitudinal studies of genetic high risk samples have reported decreases in thalamic, superior frontal and hippocampal volumes following exposure to cannabis over a 2-year follow-up period [17, 41]. Longitudinal data in first-episode schizophrenia has indicated cortical thinning in several regions known to share topographic connections with the thalamus including lateral PFC and anterior cingulate cortex [42], in addition to accelerated gray matter volume loss and ventricular enlargement [43], following exposure to cannabis during a 5-year follow-up period. Taken together, the literature suggests that people at CHR of psychosis and people with schizophrenia may have a particular sensitivity to brain tissue loss after exposure to cannabis. However, the current findings indicate that in CHR youth current cannabis use does not result in functional dysconnectivity in thalamic circuits.

Evaluation of CHR with a moderate/high-frequency of cannabis use compared to those with low-use frequency and abstinent CHR indicated no differential effects of cannabis use frequency on thalamic connectivity. It should be noted that our sample had only one high-frequency user, precluding evaluation of the effects heavy cannabis use on thalamic neural circuitry. Recent work by Smith et al. [44] demonstrated that former heavy cannabis users (who were otherwise psychiatrically healthy) show cannabis-related morphological differences in the thalamus that were consistent with and more pronounced in schizophrenia subjects who were former heavy users. Evaluation of thalamic connectivity in CHR heavy cannabis users may be more informative. The present results suggest that thalamic dysconnectivities previously reported in this population [7] do not reflect cannabis use frequency patterns.

Several limitations should be noted. The ascertainment of cannabis use relied on participants’ self-report, which may be less reliable than collection of biologically based measures such as urine toxology data. Details on cannabis dosage were not collected and therefore their potential impact on thalamic connectivity cannot be determined. It should also be recognized that the current findings offer little insight into the cellular processes that result in the observed correlation between thalamo-cortical function and age at onset of cannabis use. Furthermore, cortical regions appear to be connected to distinct, non-overlapping regions of the thalamus [2], and the current analysis did not parcellate the thalamus into distinct nuclei. Although the current study focused on the thalamus, both CHR and chronic patients with schizophrenia have been characterized by alterations across striatal and cortical networks [45–49], and the effects of cannabis use on connectivity in these circuits has not been established. The striatum may be particularly sensitive to cannabis use, as there is some evidence for increased sensitivity to delta-9-tetrahydrocannabinol, the main psychoactive ingredient of cannabis, in striatal subregions in people at genetic high risk for psychosis and in people with psychotic disorders [50]. Also, it remains unknown whether thalamic connectivity varies over time with cannabis consumption, and there is some longitudinal data in genetic high risk samples indicating decreases in thalamic volumes following exposure to cannabis over a 2 year period [17, 41]. In this regard, longitudinal evaluation of thalamic connectivity and its covariation with cannabis use patterns may also be relevant in CHR samples. There is limited research on cannabis use and brain structure and function in CHR samples; therefore, replication is needed to determine generalizability of the findings.

## Conclusions

In summary, the current work found a significant correlation between attenuated thalamo-cerebellar connectivity and a younger age at onset of cannabis use in our CHR sample. CHR who used cannabis before age 15 did not differ on thalamic connectivity as compared to CHR who used after age 15 or CHR who were cannabis naïve. No group differences in thalamic connectivity emerged when comparing CHR moderate/high-frequency users to low-frequency users or naïve CHR. These findings suggest that a younger age at onset of cannabis use is associated with disrupted coupling between the thalamus and left sensory/motor cortex. However cannabis use does not appear to be an identifying characteristic for thalamic connectivity in CHR who used before age 15, or in moderate/heavy users compared to low-frequency users or those who are abstinent.

## Additional files

Additional file 1:
**Contains details on neuroimaging data acquisition, preprocessing and analysis.** (DOCX 31 kb) Additional file 2:
**Contains demographic and clinical characteristics of CHR separated by age at onset of cannabis use and controls.** This includes sex, race, cannabis, alcohol and tobacco usage, as well as age, education and positive symptom severity. (DOCX 22 kb) Additional file 3:
**Contains demographic and clinical characteristics of CHR separated by cannabis use frequency and controls.** This includes sex, race, cannabis, alcohol and tobacco usage, as well as age, education and positive symptom severity. (DOCX 22 kb)
