# Supplementary material for: Evaluating the impact of cannabis use on thalamic connectivity in youth at clinical high risk of psychosis
Source: BMC Psychiatry. 2015 Nov 9;15:276. doi: 10.1186/s12888-015-0656-x (PMC4640353; doi:10.1186/s12888-015-0656-x)
Supplement: Additional file 1: — Contains details on neuroimaging data acquisition, preprocessing and analysis. (DOCX 31 kb) [file 12888_2015_656_MOESM1_ESM.docx]

Supplement 1

*Neuroimaging Data Acquisition*

Scanning was performed at eight sites. Five sites (UCLA, Emory, Harvard, UNC, and Yale) used Siemens-Trio 3T scanners, two sites (Zucker-Hillside Hospital and UCSD) used GE HDx scanners, and one site (Calgary) used a GE Discovery scanner. Despite scanner differences, functional imaging protocols were identical across sites, and there were no differences when ‘scanner’ was included as a covariate in the analyses. Functional images sensitive to blood-oxygenation-level-dependent (BOLD) signal were collected at all sites with axial slices parallel to the anterior-posterior commissure (AC-PC) line using a T2*-weighted gradient-echo, echo-planar sequence (TR/TE=2000/30ms, flip angle=77°, field of view=22x22cm, acquisition matrix=64x64, voxel size=3.4x3.4x4.0mm), ensuring whole-brain coverage. Functional data collection lasted 5.13 minutes, resulting in 154 volumes (30 slices/volume, inter-slice gap=1mm). Subjects were instructed to remain awake in the scanner and keep their eyes open. Subjects were excluded if they fell asleep or if their head movement exceeded 1mm along any axis. Structural images on the Siemens-Trio 3T scanners were acquired using a T1-weighted, 3D magnetization-prepared rapid gradient-echo (MPRAGE) sequence in the sagittal plane (TR/TE/TI=2300/2.91/900ms, flip angle=9°, voxel size=1.0x1.0x1.2mm, field of view=256x240x192mm). Structural images on the GE scanners were acquired using a T1-weighted, 3D spoiled gradient echo (IR-SPGR) sequence in the sagittal plane (TR/TE/TI=7.0/minimum full/400ms, flip angle=8°, voxel size=1.0x1.0x1.2mm, field of view=26cm). All structural images were inspected for quality by a trained rater, blind to group assignment (AA).

*Neuroimaging Data Preprocessing & Analysis Details*

All preprocessing followed best practices in the clinical connectivity literature ([Anticevic et al., 2012a](#_ENREF_7); [Cole et al., 2011](#_ENREF_19)). We performed the following steps: i) slice-time correction, de-banding and normalization to whole brain mode 1000, ii) removal of first 5 images from each run, iii) rigid body motion correction, iv) 12-parameter affine transform of the structural image to the Talairach coordinate system, and v) co-registration of volumes to the structural image with 3x3x3mm re-sampling. Furthermore, we employed the following rigorous quality assurance criteria for each participant to ensure comparable BOLD quality: i) signal-to-noise ratios (SNR)>100; SNR was computed by obtaining the mean signal and standard deviation (sd) for a given slice across the BOLD run, while excluding all non-brain voxels across all frames ([Anticevic et al., 2012a](#_ENREF_7)); ii) no BOLD run with a single frame movement greater than 1 functional voxel; iii) all BOLDs were movement-scrubbed ([Power et al., 2012](#_ENREF_37), [2013](#_ENREF_38)), and subjects with more than 50% frames flagged as potentially affected by movement artifacts were completely excluded from analyses. First, frames in which collective displacement across all 6 rigid body movement correction parameters exceeded 0.5mm (assuming 50mm cortical sphere radius) were identified. Second, root mean square (RMS) of differences in intensity between the current and prior frame was computed across all voxels, divided by mean volume intensity and normalized to time series median. Frames in which normalized RMS>1.6 were identified. We excluded frames flagged by either criterion, and the one frame proceeding and two frames following each flagged frame were also removed. Subjects with >50% frames flagged were omitted from analyses.

After these criteria were implemented, there were no between-group differences in SNR. The proportion of scrubbed frames differed slightly across groups. Importantly, the two clinical groups displayed no between-group differences in the proportion of scrubbed frames. Nevertheless, we ensured that the proportion of removed frames did not significantly relate to reported effects by using the proportion of scrubbed frames as a covariate across reported analyses, which did not alter effects.

Next, we removed additional potentially spurious signal in resting-state data, as is standard practice ([Biswal et al., 2010](#_ENREF_14)). All images were spatially smoothed using a 6mm full-width-at-half-maximum (FWHM) Gaussian kernel and then underwent high (>0.009 Hz) and low (<0.08 Hz) pass temporal filtering, removal of nuisance signal from ventricles and deep white matter, removal of global mean signal (GMS), and 6 rigid-body motion correction parameters plus their first derivatives using in-house Matlab tools ([Repovs et al., 2011](#_ENREF_42)). All nuisance regressors (i.e. ventricles, deep white matter and global mean signal) were defined via FreeSurfer software ([Fischl et al., 2002](#_ENREF_23)), based on individual subjects’ anatomically-based segmentations (visually inspected for quality by a trained rater, AA). Given recent reports that GMS removal can complicate analyses in cases of greater whole-brain variability ([Yang et al., 2014](#_ENREF_54)), we repeated the core analyses without removing GMS; effects remained unchanged, consistent with two recent reports that GMS removal does not significantly affect overall thalamo-cortical findings in schizophrenia ([Yang et al., 2014](#_ENREF_54)).
