# Supplementary material for: Evaluating the impact of cannabis use on thalamic connectivity in youth at clinical high risk of psychosis
Source: BMC Psychiatry. 2015 Nov 9;15:276. doi: 10.1186/s12888-015-0656-x (PMC4640353; doi:10.1186/s12888-015-0656-x)
Supplement: Additional file 3: — Contains demographic and clinical characteristics of CHR separated by cannabis use frequency and controls. This includes sex, race, cannabis, alcohol and tobacco usage, as well as age, education and positive symptom severity. (DOCX 22 kb) [file 12888_2015_656_MOESM3_ESM.docx]

Additional file 3: Table S3. Demographic and clinical characteristics of CHR separated by current cannabis use frequency and controls.

|  | **Abstinent**  **n=529** | **Low- frequency**  **n=82** | **Moderate-frequency**  **n=27** | **High-frequency**  **n=20** |  |  |  |
| --- | --- | --- | --- | --- | --- | --- | --- |
|  | **n (%)** | **n (%)** | **n (%)** | **n (%)** | **χ ^2^** | ***df*** | ***p*-value** |
| Sex |  |  |  |  |  |  |  |
| Male  Female | 12 (46)  24 (54) | 20 (83)  4 (17) | 64 (57)  48 (43) | 57 (54)  48 (46) | 8.4* | 3 | 0.04 |
| Race |  |  |  |  |  |  |  |
| First Nations  Asian  Black  Latin America/Middle East/White  Inter-racial | 0 (0)  0 (0)  1 (4)  22 (84)  3 (12) | 0 (0)  1 (4)  6 (25)  14 (58)  3 (13) | 2 (2)  8 (7)  28 (25)  57 (51)  17 (15) | 2 (2)  10 (10)  28 (27)  59 (55)  6 (6) | 26.8 | 24 | 0.32 |
| AUS/DUS cannabis use severity |  |  |  |  |  |  |  |
| Abstinent  Use without impairment  Abuse | 4 (15)  20 (77)  2 (8) | 0 (0)  17 (71)  7 (29) | 112 (100)  0 (0)  0 (0) | 92 (88)  13 (12)  0 (0) | 201.2^A,B^ | 6 | <0.001 |
| AUS/DUS alcohol use severity |  |  |  |  |  |  |  |
| Abstinent  Use without impairment  Abuse  Dependence | 5 (19)  21 (81)  0 (0)  0 (0) | 1 (4)  21 (88)  2 (8)  0 (0) | 75 (67)  33 (29)  3 (3)  1 (1) | 60 (57)  45 (43)  0 (0)  0 (0) | 53.7^B,C,D^ | 9 | <0.001 |
| AUS/DUS tobacco use severity |  |  |  |  |  |  |  |
| Abstinent  Use without impairment  Abuse  Dependence | 13 (50)  13 (50)  0 (0)  0 (0) | 8 (32)  14 (58)  2 (8)  0 (0) | 94 (84)  16 (14)  2 (2)  0 (0) | 94 (89)  11 (11)  0 (0)  0 (0) | 69.5^A,B^ | 9 | <0.001 |
|  | **Mean (SD)** | **Mean (SD)** | **Mean (SD)** | **Mean (SD)** | **F** | ***df*** | ***p*-value** |
| Age (years) | 19.0 (3.3) | 20.1 (3.3) | 19.4 (4.6) | 19.5 (4.6) | 0.3 | 3, 263 | 0.82 |
| Education (years) | 12.0 (2.7) | 12.3 (2.3) | 11.7 (2.5) | 12.7 (3.5) | 2.1 | 3, 262 | 0.11 |
| SOPS total attenuated positive symptoms | 12.2 (3.9) | 13.5 (4.2) | 11.8 (3.7) | 1.2 (1.7) | 256.0^E^ | 3, 263 | <0.001 |

*Moderate/High-frequency had more males than the abstinent and low-frequency groups

^A^Low-frequency, Moderate/High-frequency and Controls > Abstinent

^B^Moderate/High-frequency and Low-frequency > Controls

^C^Low-frequency and Moderate/High-Frequency > Abstinent

^D^Moderate/High-frequency > Low-frequency

^E^Abstinent > Controls
